# Effect of classical laryngeal mask or I-gel use on otolaryngeal system in ambulatory inhalation general anesthesia: A prospective study

**DOI:** 10.12669/pjms.40.10.8416

**Published:** 2024-11

**Authors:** Sedat Saylan, Hatice Bengu Yaldiz Cobanoglu

**Affiliations:** 1 Dr. Sedat Saylan, Department of Anesthesiology and Intensive Care Medicine, Faculty of Medicine, Karadeniz Technical University, Trabzon, Turkiye; 2 Dr. Hatice Bengu Yaldiz Cobanoglu, Department of Otorhinolaryngology, Faculty of Medicine, Karadeniz Technical University, Trabzon, Turkiye

**Keywords:** cLMA, I-gel, Otolaryngeal system, Ambulatory surgery, General anesthesia

## Abstract

**Objective:**

To investigate the effects of supraglottic airway tools such as classical laryngeal mask (cLMA) and I-gel, which can be used without the need for muscle relaxation in the airway management of general anesthesia patients, on the otolaryngeal system.

**Methods:**

This prospective randomised study was conducted at Karadeniz Technical University Hospital, Faculty of Medicine, Trabzon, Turkey, during November 2020 to December 2021. Eighty-nine patients in the American Society of Anesthesiologists (ASA) grade I-II group, who would undergo elective surgery under general anesthesia, were randomized into two groups, namely Group cLMA and Group I-gel. cLMA and I-gel were used for airway management of the patients. After anesthesia induction, tympanometric measurements were taken at regular intervals for middle ear pressures of both ears.

**Results:**

While air way pressures, SpO_2_ and EtCO_2_ values were within normal limits, there were no differences in terms of complications. In tympanometric measurements, middle ear pressure increase was statistically higher in the cLMA group than in the I-gel group (p <0.001).

**Conclusions:**

We think that I-gel may be a more advantageous supra glottic airway device in terms of otolaryngeal effecs and middle ear pressure in theair way management during short surgical procedures.

## INTRODUCTION

Ambulatory surgery allows the patient to return home on the same day; reduces the hospital occupancy rate, costs, and risk of infection; and facilitates early return to social and professional activities.^1^ Inhalation anesthetics continue to be the most popular option for maintaining daily anesthesia, as they are always ready on the anesthesia devices in the operating room, are easy to carry and control, and exert their effects quickly. However, inhalation anesthetics pass through the eustachian tube to the middle ear, causing changes in the middle ear pressure (MEP). Pressure changes in the middle ear can cause tympanic membrane rupture, hemotympanum, and permanent or temporary hearing loss.^2^

In short surgical interventions, supraglottic airway devices (SADs) providing an airway that is less invasive than intubation but safer than a ventilation mask has been implemented to maintain airway patency after anesthesia induction.^3^ The cLMA is the most commonly used SAD, and has an inflatable cuff that is invasive, anatomically placed between the facemask and the endotracheal tube and is generally inserted at the larynx level to preserve spontaneous respiration.^4^

SADs such as the classical laryngeal mask (cLMA, Teleflex Medical Europe Ltd, County Westmeath, Ireland) and I-gel (Intersurgical Ltd., Wokingham, Berkshire, UK) are safely used in the daily airway management of general anesthesia patients without the need for muscle relaxation. These airway tools reduce the complications related to endotracheal intubation (ETI) while providing ease of application.

This study investigated the effect of two different SADs on MEP, and changes in the upper respiratory tract under general inhalation anesthesia. This study aimed to investigate the effects of supraglottic airway tools such as classical laryngeal mask (cLMA) and I-gel, which can be used without the need for muscle relaxation in the airway management of general anesthesia patients, on the otolaryngeal system.

## METHODS

This prospective randomised study was conducted at Karadeniz Technical University Hospital, Faculty of Medicine, Trabzon, Turkey, during November 2020 to December 2021. This study included patients scheduled for knee arthroscopy under general anesthesia (minor orthopedic surgery for less than one hour) and provided the safety of airway access with cLMA or I-gel. Written informed consent was obtained from all the included patients.

### Ethical Approval:

The permission for the study was obtained from the institutional ethics committee (2017/128, date July 28, 2017),

We included patients in the American Society of Anesthesiologists (ASA) classification grade I-II group aged 18–65 years whose surgery duration did not exceed 60 minutes. Patients with any anatomical disorders in the upper airway, adenotonsillar hypertrophy, perforated tympanic membrane, a history of tympanoplasty surgery or tympanic membrane perforation, respiratory diseases such as asthma and COPD, difficult and traumatic intubation, or obesity (BMI≥30 kg/m^2^) were excluded from the study. Patients were included only when no otorhinolaryngologic abnormalities were detected on examination by a national board-certified ear, nose and throat (ENT) doctor. The patients whose written consent was obtained and preoperative evaluations were made were randomly divided into cLMA (n=44) and I-gel (n=45) groups by computer-assisted randomization method.

Baseline clinical data were recorded by performing routine monitoring (electrocardiography, noninvasive blood pressure measurement, peripheral oxygen saturation) in the operation room without any premedication. Tympanometric measurements of both ears were recorded with noninvasive interaoustics MT-10 tympanometry device.

Induction anesthesia was performed in all patients with 1 mcg kg^-1^ fentanyl, 1 mg kg^-1^ lidocaine, and 2–3 mg kg^-1^ propofol as induction agents after 2mg midazolam. No neuromuscular agents were used. Anesthesia was maintained with a mixture of 2% sevoflurane and N_2_O/O_2_:60:40 in controlled mechanical ventilation with a tidal volume of 6–8 mL kg^-1^ at 12 breaths/minute.

Patients who were ventilated with 100% O_2_ for two minutes randomly with an appropriate-size mask, I-gel (no: 3,4,5) or c LMA (no: 4,5) depending on the weight, were applied by a nonparticipant anesthesiologist. Lubricants (water-based gel) were applied to the outer surfaces of all I-gels and cLMAs prior to placement. The cLMA cuff was inflated with the recommended volume of air. The cLMA cuff pressure was checked with a pressure gauge (VBM Medizintechnik GmbH, Germany) at 60cm H_2_O. The appearance of bilateral chest movements with at least 6 mL/kg tidal volume accompanied by a square wave capnograph was considered sufficient for successful intervention and effective ventilation.

Patients’ tympanometric measurements for both ears were recorded preoperatively, after the insertion of SADs at 5, 10, and 30 minutes, right after the removal of SAD, and at 30 minutes postoperatively. Tympanometric functions were evaluated between -400 decapascals (daPa) and +200 daPa. Middle ear pressure, outer canal volume, gradient, and acoustic reflexes for each ear were measured by an ENT specialist who was blinded to the type of SAD. Possible complications such as nausea, vomiting, dysphagia, laryngospasm, postoperative otalgia, postoperative sore throat, hearing loss, hemotympanum, hoarseness, and bloodstain of the SADs were recorded. After spontaneous breathing was regular and sufficient, extubation was performed. After six hours follow-up, patients were discharged from the hospital after becoming fully conscious, able to communicate, and have a good intake of oral nutrition without any complaints.

### Statistical analysis:

The power analysis was performed for two groups in the sample size study. When the effect size was considered as Cohen d=0.6, 80% power would be obtained by including 90 patients in the study (α=0.05) (G. Power version 3.9.1.2, Germany). The data were analyzed with SPSS version 23.0. We analyzed descriptive statistics. Normal distribution of numeric variables was evaluated by the Kolmogorov-Smirnov test. We presented numeric variables as mean ± standard deviation and categorical variables as noun (% percent). Independent samples t test was used in binary comparisons of independent variables. To compare dependent variables, paired t-test was used. The chi-square test was used to compare categorical variables. The statistical significance level was set as P<0.05.

### Patients consent:

All participants provided in formed consent before taking part in the study.

## RESULTS

**Fig.1:**
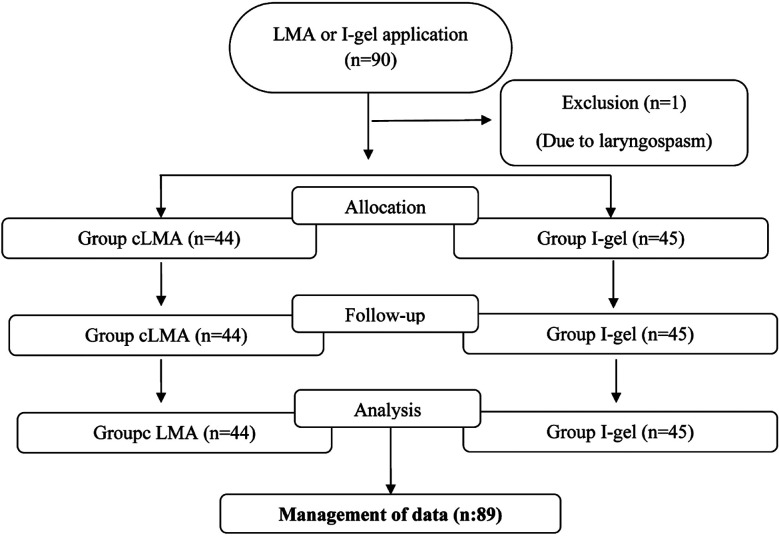
Flow chart of the study.

A total of 89 patients, 44 in the cLMA group and 45 in the I-gel group, were included in the study. One patient was excluded because of the development of laryngospasm on intubation (Fig.1). No significant differences were noted between the groups in terms of age, sex, ASA scores, BMI, duration of anesthesia, and operation time (Table-I), indicating homogeneity and randomization of the groups.

**Table-I:** Demographic and main parametric data.

	cLMA (n=44)	I-gel (n=45)	p
Age [year, (mean±SD)]	39.4±11.1	39.3±13.7	0.971*
Gender [female/male, n (%)]	22(50%) / 22(50%)	14(31.1%) / 31(68.9)	0.069**
Weight (kg)	73.7±10.9	72.3±11.2	0.542*
Height (cm)	170.8±7.5	170.7±7.1	0.922*
Body Mass Index (kg/cm^2^)	25.1±2.3	24.7±2.9	0.508*
ASA Physical Status (I/II)	27(61.4%) / 17(38.6%)	21(46.7%) / 24(53.3%)	0.164**
Duration of anesthesia (minute)	57.1±2.2	57.4±3.7	0.667*
Duration of surgery (minute)	45.6±2.6	44.4±2.5	0.330*

ASA: American Society of Anesthesiologist,

* Chi square test

** Independent samples t test.

Mean airway pressure, peak airway pressure, SpO2, and EtCO2 values were within normal limits in all patients and were not significantly different between the groups. The most frequent complication was dysphagia in the cLMA group and nausea in the I-gel group. There was no laryngospasm, otalgia, hemotympanum, hearing loss, or hoarseness. There was no significant difference between the groups in terms of complications such as nausea, vomiting, dysphagia, postoperative sore throat, and bloodstain of the SADs (Table-II).

**Table-II:** Intraoperative and postoperative complications of both group.

	LMA (n=44)	I-gel (n=45)	p*
Postoperative nausea-vomiting	4(9.1%)	3(6.7%)	0.671
Dysphagia	6(13.6%)	2(4.4%)	0.130
Laryngospasm	0	0	NA
Postoperative otalgia	0	0	NA
Postoperative sore throat	5(11.4%)	1(2.2%)	0.086
Hemotympanum	0	0	NA
Hoarseness	0	0	NA
Bloodstain of the SAD	5(11.4%)	1(2.2%)	0.086

* Chi square test.

**Fig.2:**
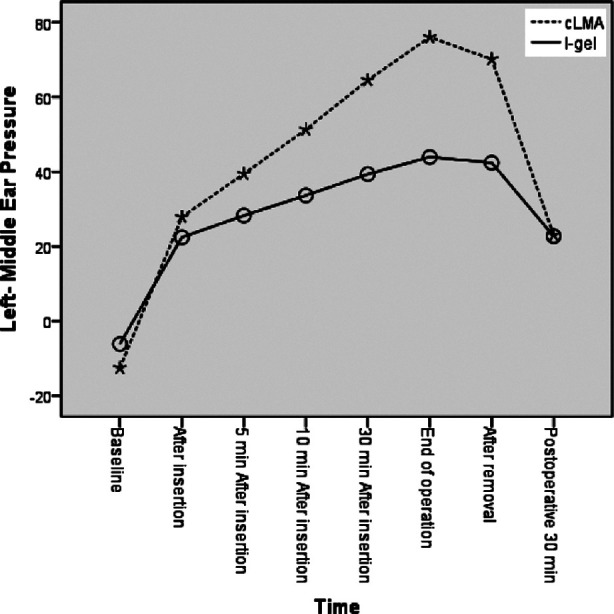
Middle ear pressure (left).

In tympanometric measurements, a significant difference was observed between the cLMA and I-gel groups. From the application until removal, all measurement changes were statistically significant for left-middle ear pressure. The measurement changes for 10^th^, 30^th^ minutes after insertion, end of operation, and after removal were statistically significant for right-middle ear pressure. There was no difference between groups at 30 minutes postoperatively. The middle ear pressure was significantly lower in the I-gel group during the operation (Table-III, Fig.2).

**Table-III:** The Middle Ear Pressures.

	Right- MiddleEarPressure			Left- MiddleEarPressure		

	cLMA	I-gel	p**	cLMA	I-gel	p**
Baseline	-11.18±17.16	-9.96±23.49	0.21	-12.57±14.56	-6.11±18.44	0.71
After insertion	27.20±10.37	25.47±11.93	0.46	27.89±8.66	22.42±7.85	<0.001
5 min Afterinsertion	38.77±10.65	32.67±16.35	0.04	39.43±9.37	28.27±8.12	<0.001
10 min Afterinsertion	53.64±10.91	38.91±16.07	<0.001	51.30±10.54	33.67±9.47	<0.001
30 min Afterinsertion	66.16±11.17	43.93±15.15	<0.001	64.52±12.30	39.36±10.08	<0.001
End of operation	75.59±12.17	48.89±15.84	<0.001	76.05±14.11	43.93±10.71	<0.001
After removal	69.27±13.05	47.22±14.02	<0.001	70.11±16.81	42.40±11.15	<0.001
Postoperative 30 min	24.09±8.962	25.62±10.54	0.46	23.02±7.599	22.71±8.20	0.85

** Independent samples t test.

**Fig.3:**
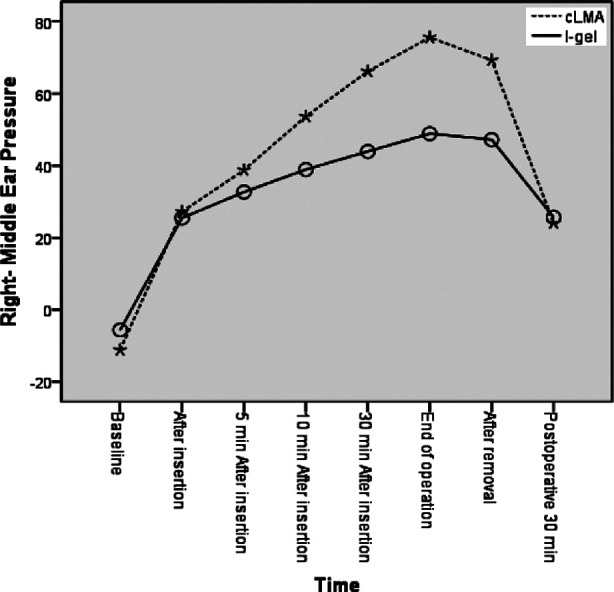
Middle ear pressure (right).

## DISCUSSION

This study was performed in patients who underwent knee arthroscopy surgery under general anesthesia with two types of SADs used for airway safety. Inhalation anesthetics continue to be the most popular option for maintaining anesthesia. Sevoflurane has low solubility, allowing a rapid increase in anesthetic concentration; also, it does not irritate the upper airways and provides easy control and termination, making it advantageous in routine anesthesia.^5^ Nitrous oxide (N_2_O) is a colorless, odorless gas and has been used in anesthesia practice for more than 150 years. Its onset of action is fast. Although it does not provide complete anesthesia on its own, it provides a synergistic effect when used with other inhalation or intravenous agents. N_2_O improves the quality and safety of anesthesia induction, facilitates faster recovery, and reduces overall costs, thereby maintaining its place in modern daily anesthesia.^6^ All these positive features led us to prefer these two inhalation agents for the maintenance of anesthesia in our daily patients.

Although ETI is the gold standard in airway management, supraglottic tools such as cLMA and I-gel ensure airway safety in appropriate cases while minimizing ETI-related complications. It can also be applied safely and easily in experienced hands without the need for muscle relaxants.^7^,^8^ We compared two SADs in our cohort during routine surgery to plan discharge on the same day and to avoid possible complications of ETI. We successfully used them in all patients without using any neuromuscular agents. Only one patient in the cLMA group needed neuromuscular blockers due to the development of laryngospasm, and this patient was excluded from the study.

Regarding postoperative complications, none of the patients developed laryngospasm, postoperative otalgia, hoarseness, hemotympanum, or hearing loss. However, 6 (13.6%) patients in the cLMA group and 2 (4.4%) in the I-gel group developed dysphagia, and 5 (11.4%) and 1 (2.2%), respectively, developed postoperative sore throat. Chauhan et al. reported that LMA ProSeal caused sore throat more frequently than I-gel. The authors attributed postoperative complications to trauma during placement, multiple interventions, and the pressure of the cuff on the pharyngeal mucosa.^9^ Although Bindal et al.^10^ did not report a significant intergroup difference in postoperative dysphagia and sore throat, they stated that severe dysphagia and severe sore throat were higher in the cLMA group than in the I-gel group. Similarly, we too observed no difference between the groups in postoperative dysphagia and sore throat, the frequency was higher in the cLMA group. When our study was evaluated in terms of complications, there was no significant difference between the two groups and it was similar to previous studies.^11^,^12^ Allahyari et al.^13^ in their study, stated that the frequency of postoperative nausea and vomiting in the I-gel group was significantly lower than the cLMA and ETT groups. In our study, there was no significant difference between the groups in the rate of postoperative nausea and vomiting, which is an important postoperative complication.

In our study, bloodstain was observed in 5 (11.4%) patients in the cLMA group and 1 (2.2%) in the I-gel group in the mask examination performed after removal of SAD at the end of the operation. Classic LMAs have a higher rate of bloodstains due to their inflatable cuffs. In the literature, the incidence of bloodstains for I-gel is 2%–6% and for cLMAis 12%–36%, which is close to our results. Notably, although bloodstain was detected in more patients in the cLMA group, the difference was not significant.^14^,^15^

The eustachian tube opens during swallowing and chewing, allowing nitrogen to go to the middle ear and balance the middle ear pressure. Therefore, it is a voluntary movement. The relaxation of palatal muscles during anesthesia leads to a delay in the equalization of middle ear pressure with atmospheric pressure.^16^ Sudden changes in middle ear pressure can lead to symptoms such as otalgia, otitis media, nausea, and vomiting. Deniz et al.^17^ examined the effects of inhalation general anesthetics on MEP and found that sevoflurane affects MEP the least. The authors stated that sevoflurane can be used safely in middle ear surgeries with EtCO2 and airway pressure monitoring. Hohlrieder et al.^18^ concluded that different airway devices, with or without N_2_O, did not affect the MEP. However, they did not use I-gel in that study. In our study, no significant difference between EtCO2 levels and airway pressures was found between the two groups, which, we believe, is critical to accurately show the effects of the inhalation agents on MEP.

Tympanometry is an objective, easily accessible, portable, and noninvasive measurement used to evaluate changes in middle ear pressure. The measurements can be made in a few minutes through a probe placed in the external ear canal of the patient. In tympanometric measurements, peak middle ear pressure values between -100 and +50 daPa are considered normal.^19^ Many studies on rhinological and other otorhinolaryngologic surgeries have used tympanometry to measure intraoperative middle ear pressure.^20^ Patient position, inhalation, intravenous anesthetic agents, and airway pressures affect middle ear pressure. Therefore, we evaluated the same surgical group of patients in the same position to measure the pressures. We used the same anesthetic agents in all patients to eliminate this limitation. Although studies have assessed the relationship between middle ear pressure and different anesthetic gases or different intubation devices, no study has compared SADs in routine surgical procedures with respect to middle ear pressure.^21^,^22^

### Limitations:

We conducted our study in the low-risk group (ASA class I and II) and in patients with normal airway. We did not use a flexible fiberoptic bronchoscope to evaluate the anatomical position of the SADs. We did not compare it with other SADs and endotracheal tube because the cLMA and I-gel are the most frequently used SADs in the clinic.

## CONCLUSION

In this study, two SADs were compared in terms of middle ear pressure and complications. Our findings revealed that I-gel was significantly superior to LMA in normalizing middle ear pressure values, although there was no difference in terms of complications. This may be attributed to the structure of I-gel it can be placed more easily or perhaps the gel layer may have a lower effect on pressure. I-gel was found to have minimal effect on middle ear pressure. Further research is required to determine the best SADs for patients with otologic pathology if necessary.

### Authors` contribution:

**SS** conceived, designed and did statistical analysis & editing of manuscript, is responsible for integrity of research.

**HBYC** did data collection, Review and manuscript writing
